# Screening of biocontrol bacteria against soft rot disease of *Colocasia esculenta* (L.) *schott* and its field application

**DOI:** 10.1371/journal.pone.0254070

**Published:** 2021-07-12

**Authors:** Xiaofei Dong, Lu Fang, Zuyun Ye, Guangqiang Zhu, Qianyu Lai, Shengrong Liu

**Affiliations:** 1 Ningde Normal University, Fujian province, China; 2 The Engineering Technology Research Center of Characteristic Medicinal Plants of Fujian, Ningde Normal University, Fujian province, China; Fujian Agriculture and Forestry University, CHINA

## Abstract

Soft rot disease is a major pathogenic bacteria of Fuding areca taro and has caused serious losses. This study aims to screen biocontrol bacterial against soft rot disease. A total of 53 bacterial strains were isolated from the rhizosphere soil, nine of which exhibited good biocontrol effect against the pathogenic bacteria of soft rot disease as seen in antagonistic screening of biocontrol bacteria from corm in vitro. Strains were selected by physical and chemical experiments, biocontrol effect tests in vivo, molecular sequencing, morphological observation and field tests. Four strains including CAB-L005, CAB-L012, CAB-L014, and CAB-L022 exhibited strong antagonistic effects. On the basis of the sequence homology of 16S rRNA genes, the similarity between strain CAB-L005 and *Bacillus tropicus* was 100%, that between strain CAB-L012 and *Bacillus subtilis* was 99%, and that between strain CAB-L014 and *Bacillus tequilensis* was 100%, and similarity between strain CAB-L022 and *Bacillus cereus* was 100%. The isolated bacteria demonstrated good biocontrol effects in field experiments. In this study, four strains with good biocontrol application value were isolated and identified, providing a foundation for biocontrol against soft rot disease in areca taro.

## Introduction

Areca taro (*Colocasia esculenta* (L.) *schott*) is a perennial herb belonging to the *Araceae* family. It is nutritionally rich and tasty with high market value [1]. The Ministry of Agriculture of the People’s Republic of China approved the registration and protection of agricultural geographical areas for growing “Fuding areca taro” on October 22^nd^, 2011. At present, with agricultural lands covering an area of more than 2,000 hectares, the planting industry of areca taro is the pillar industry of Fuding agriculture with more than $42 million worth of crop being produced. Soft rot disease is one of the main issues affecting the production of areca taro. The incidence of soft rot disease in the plant is generally 3 to 15%, and in severe cases exceeds 50% or even leads to failure of harvest, which hurts the enthusiasm of taro farmers [2]. Soft rot disease is caused by *pectobacterium carotovorum subsp*. *Carotovorum*, a pathogenic bacteria [3]. This disease mostly occurs at the base of the corm or petiole. The pathogen overwinters in soil or corm, invades the plant through a wound or the stoma, then reinfects the plant through an insect vector or irrigation. The disease was more likely to spread in high temperature, high humidity and rainy continuous cropping land. When the soft rot disease occurs, the infected part of the plant rapidly softens and rots until the whole plant withers. This disease has a significant impact on the yield of taro and on the local economy; however, the commonly used methods such as crop rotation and pesticide usage did not exhibit desirable controlling effects. In addition, even with short periods of exposure to pesticides, they can still easily produce ecological and food safety problems [4]. Previous studies have shown that the use of antagonistic bacteria or their metabolites attached to plants or rhizosphere soil to regulate the balance of harmful microorganisms around the roots may achieve the purpose of disease control and yield conservation and is an important method to prevent and control soil borne diseases [5,6].

The guiding principles of agricultural sustainable development in China are not only to ensure food safety, develop agriculture of high-yield, high-quality, and high-efficiency, and promote the continuous growth of agricultural economy. The principles also involve the rational use of resources and the establishment of a good ecological environment in order to achieve the sustainable development of agriculture and rural areas. To form a coordinated agricultural economy, technology, ecosystem, and a sound, prosperous social system of sustainable development, the use of biological control methods for plant diseases is urgently needed [7]. Biological control is efficient and non-toxic, which can effectively retain the beneficial useful microorganisms in the environment and meet the needs of people for green food. The use of biological control microbes can effectively control diseases, Wang et al. found that *Bacillus subtilis* and *Bacillus amylolyticus* have a good effect on the control of apple tree rot disease after fermentation [8]. Chen et al. also found that *Bacillus subtilis* had a good effect on the control of tomato wilt disease [9]. Zhou et al. successfully isolated and identified biocontrol bacteria that displayed significant control against bacterial soft rot disease in *Amorphophallus konjac* from three endophytic bacteria, whose biocontrol effect reached 57%–79% [10]. Currently, the prevention and control of soft rot disease in taro consists of comprehensive technologies involving the combination of agricultural cultivation and chemical control, both of which indicate that prevention is more important than treatment [4].

At present, there are few studies on soft rot disease in areca taro. Thus, this study aims to screen biocontrol bacteria in rhizosphere soil for activity against the pathogens of soft rot disease by isolating the identified sample of soft rot disease pathogens in the early stages of the study, studying its control against soft rot disease, and applying methods in order to provide a research basis for the control of soft rot disease in areca taro.

## Materials and methods

### Soli samples and pathogenic bacteria

The soil samples were taken from the areca taro planting field of Hekeng Village, Guanling Town, Fuding City, Fujian Province (The biocontrol bacteria and areca taro, selected from the soil samples, were all taken from the farmland planted by members from the research team. Areca taro is a local species with a history of hundreds of years of cultivation, not an alien species. Endangered or protected species are not involved.). The soil samples were about 200 g each and were taken from three different rhizosphere soils of healthy plants from a depth of 10–15 cm. The pathogenic bacteria of soft rot of areca taro was *pectobacterium carotovorum subsp*. *Carotovorum*, which comed from the laboratory (strain no. 141108) study.

### Bacterial isolation

Using the method of Fang [11], 10g of soil was suspended in an Erlenmeyer flask containing 90 mL sterile water and glass beads. This was then shaken for 10 min and then diluted into soil suspensions at dilutions of 10^−1^, 10^−2^, 10^−3^, 10^−4^, 10^−5^, 10^−6^, 10^−7^, and 10^−8^. Then, 100 μL of each dilution was taken and coated evenly on nutrient agar (NA) plates in triplicates and incubated upside down in constant temperature incubators set to 37°C for 24h. Individual bacterial colonies growing on the plates were selected according to morphology, color, transparency, and other characteristics, then purified for slant cultures that were stored in a 4°C freezer.

### Screening of biocontrol bacteria from corm in vitro

Using the method of Chen et al. [12], the purified bacteria and the pathogenic bacteria of soft rot disease were inoculated in NA liquid medium then incubated in a shaking incubator for 8 h at 37°C and 220 rpm. Healthy taro were selected, washed, dried, and cut into pieces measuring 3 cm×3 cm×0.7 cm. The pieces were then sterilized with 75% ethanol for 30 s, sterilized again with 0.1% mercuric chloride for 5 min, and then washed thoroughly 5 times with sterile water. They were then each cultured onto a sterile Petri dish (9 cm in diameter) with filter paper. The liquid culture of the soft rot disease pathogens and the liquid culture of the biocontrol bacteria were mixed to a ratio of 1:1. A 1 cm×1 cm×0.3 cm cross-shaped wound was cut in the center of each piece of taro with a sterile knife and forceps. Each wound was then inoculated with 20 μL of the mixed bacterial suspension, and inoculation was repeated three times. Sterile water was used as the negative control and liquid culture of the soft rot disease pathogens as the positive control. This process was repeated 3 times, after which the tissue were incubated in constant temperature set to 37°C incubators for 7 days. Findings were observed and recorded, and photos were taken.

### Antagonistic test

Using the plate antagonistic method of Long [13], 100 μL of pathogenic bacteria suspension with a concentration of roughly 1.0×10^8^ CFU/mL was coated on a culture medium plate. Then, five discs of 5mm filter paper were soaked in the biocontrol fermentation suspension with a concentration of roughly 1.0×10^8^ CFU/mL and placed on the bacterial lawn at equal distances from each other. Filter paper soaked in sterile liquid medium was used as the negative control. Using the fermented liquid method of Gu et al. [14], 100μL of pathogenic bacteria suspension with a concentration of roughly 1.0×10^8^ CFU/mL was coated on a culture medium plate, cultured at 37°C for 24h, four holes were drilled with 8 mm diameter on the bacterial lawn at equal distances from each other, and 50μl biocontrol fermented liquid was injected. Using the sterile and fermented filtrate method of Han et al. [15], the biocontrol fermented liquid was centrifuged at 7000r/min for 10 min to remove the bacteria and take the supernatant. The supernatant was filtered by 0.22μm bacterial filter to obtain sterile fermented filtrate, and 50μl sterile fermented filtrate was injected to four holes. These plates were then cultured at 37°C and the inhibition zones of each were observed. This process was repeated three plates for each strain.

### Molecular identification

The biocontrol bacterial DNA was extracted using the bacterial genomic DNA extraction kit obtained from CWBio company. This was done using DNA sample as template and amplifying using the universal primers 27F (5’-AGTTTGATCCTGGCTCAG-3’) and 1492R (5’- GGTTACCTTGTTACGACTT-3’). The PCR Reaction System settings were as follows: one denaturation step (10 min at 94°C), 30 cycles of amplification (30s at 94°C, 30s at 55°C, 60s at 72°C), and a final elongation step of 10 min at 72°C. The PCR products were stored at 4°C. The products were sequenced by TsingKe biological company, and the sequences were used to construct a phylogenetic tree.

### Morphological examination, physiological and biochemical tests

According to the method *Manual for Systematic Identification of Common Bacteria* (Dong et al., 2001) [16].

Morphological examinnation was done via colony observation (shape, color, transparency.) and microscopic examination with gram staining, spore staining, and flagella staining.

Physical and chemical tests were aerobic, contact enzyme, glucose acid production, gelatin hydrolysis, indole, mannitol acid production, nitrate reduction, phenylalanine deaminase, starch hydrolysis, utilization of citrate salt, V-P, salinity tolerance (2%, 5%, 7%, 10%), and use of carbon and nitrogen sources (carbon sources: sucrose, citric acid, glucose. Nitrogen source: potassium nitrate.).

Growth characteristics observed were the optimum pH (pH = 5–10), the optimum temperature (4°C, 20°C, 30°C, 37°C, 41°C, 45°C), and the thermostability (65°C). These stains were then cultured at 37°C for 48h, the optical densities of the bacterial suspensions were measured by spectrophotometer every 8h (λ = 600nm). Bacterial growth was observed. This process was repeated three times.

### In vivo field experiment

The areca taro soil in the field was loosened and the soil was dug to expose the corm. Healthy corms were selected and their surfaces were cleaned. After being soaked in 75% alcohol, sterile scissors were baked over the flame of alcohol lamp. After cooling, the scissors were then inserted into the corm and rotated to form a cylindrical wound. The bacterial suspension used were the pathogenic bacteria suspension, the 1:1 mixture of pathogenic bacteria, and biocontrol bacteria suspension all with 1.0 × 10^7^ CFU/mL density. The bacterial suspensions were injected into the wounds with 2ml syringes. The wounds were then sealed with a bacterial filter membrane, covered with soil, and appropriately marked. The used scissors and syringe were not reused. Fourteen days later, the characteristics of the corm were observed. This process was repeated three times. A field experiment for biocontrol was performed on the bacteria with observed biocontrol effect. Three rows were selected from healthy plants interlacing, and the above experiments were adopted on three strains in each row.

## Results

### Bacterial isolation

Fifty-three strains of bacteria were isolated and purified from soil samples by plate streaking method. They were labeled as CAB-L001–CAB-L053.

### Screening of biocontrol bacteria from corm in vitro

**Fig 1:**
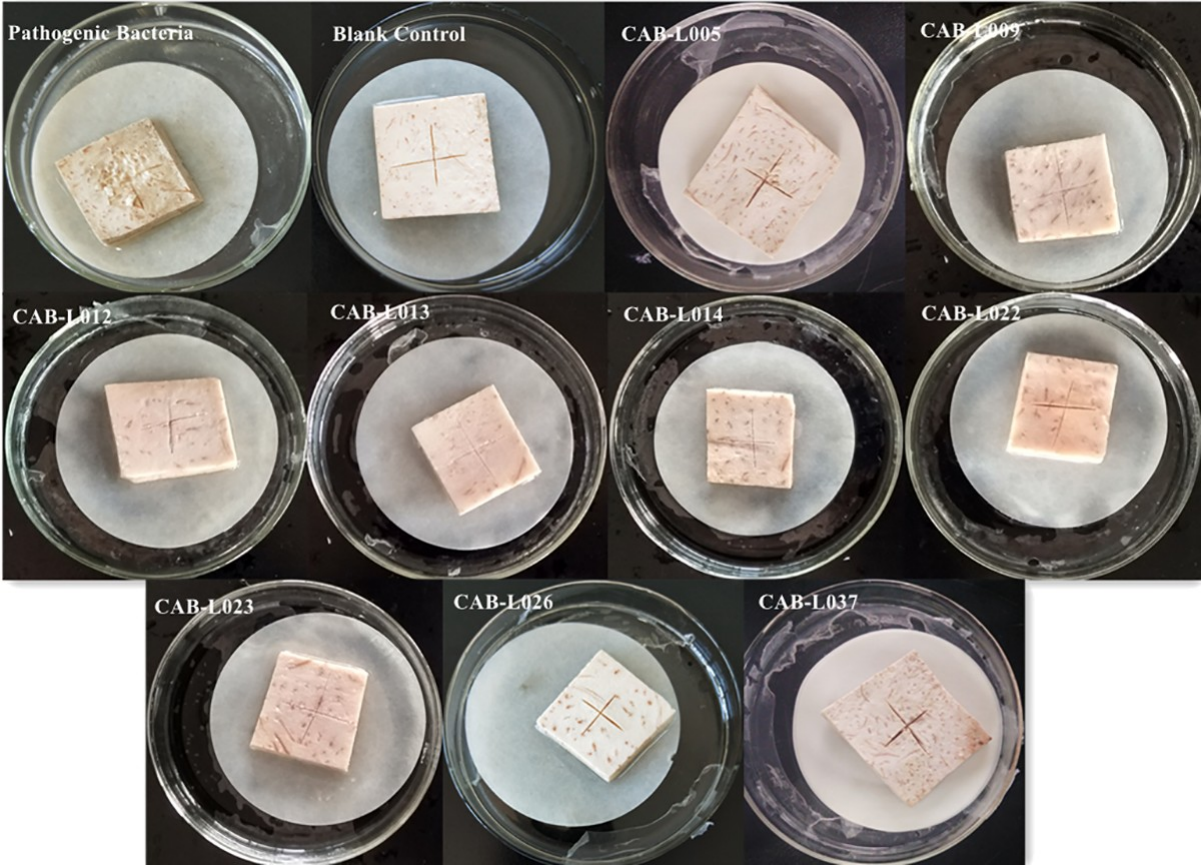
In vitro biocontrol effect of isolated bacteria on corm slices.

Among all the tissues inoculated with the 1:1 mixture of pathogenic bacteria and biocontrol bacteria, some changed color after 4 days and became rotten and foul-smelling after 7 days. The negative control group did not become rotten or foul-smelling, while the positive control group became rotten and foul-smelling. The areca taro tissue inoculated with a mixture of pathogenic bacteria and biocontrol bacteria of CAB-L005, CAB-L009, CAB-L012, CAB-L013, CAB-L014, CAB-L022, CAB-L023, CAB-L026, and CAB-L037 were not infected. However, areca taro tissues of other strains were partially or completely rotten and foul-smelling (Fig 1).

### Antagonistic test

**Fig 2:**
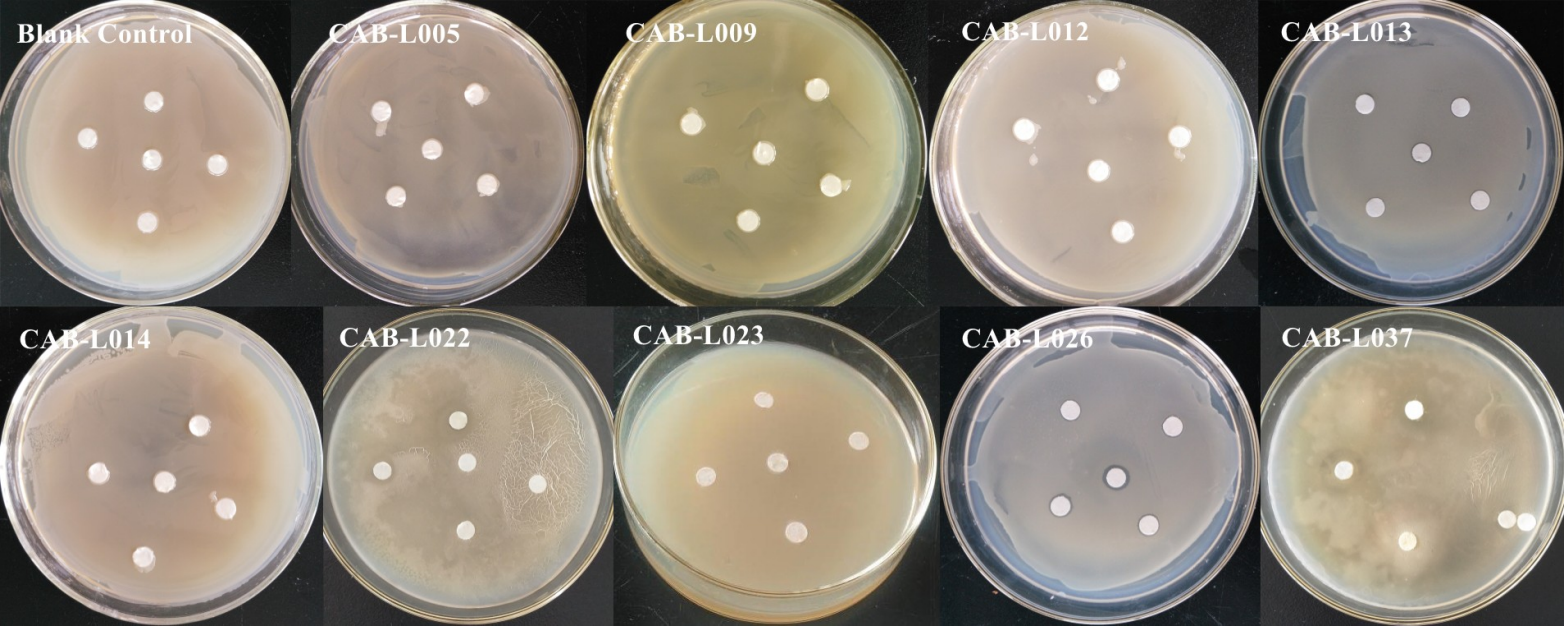
Antagonistic effect of plate.

Plate antagonistic test was carried out on the nine strains with biocontrol effect, the strain CAB-L026 had 0.5–1.5mm inhibition zones, while other strains had no inhibition zones (Fig 2).

### Antagonistic effect of fermentation broth and sterile fermentation filtrate

**Fig 3:**
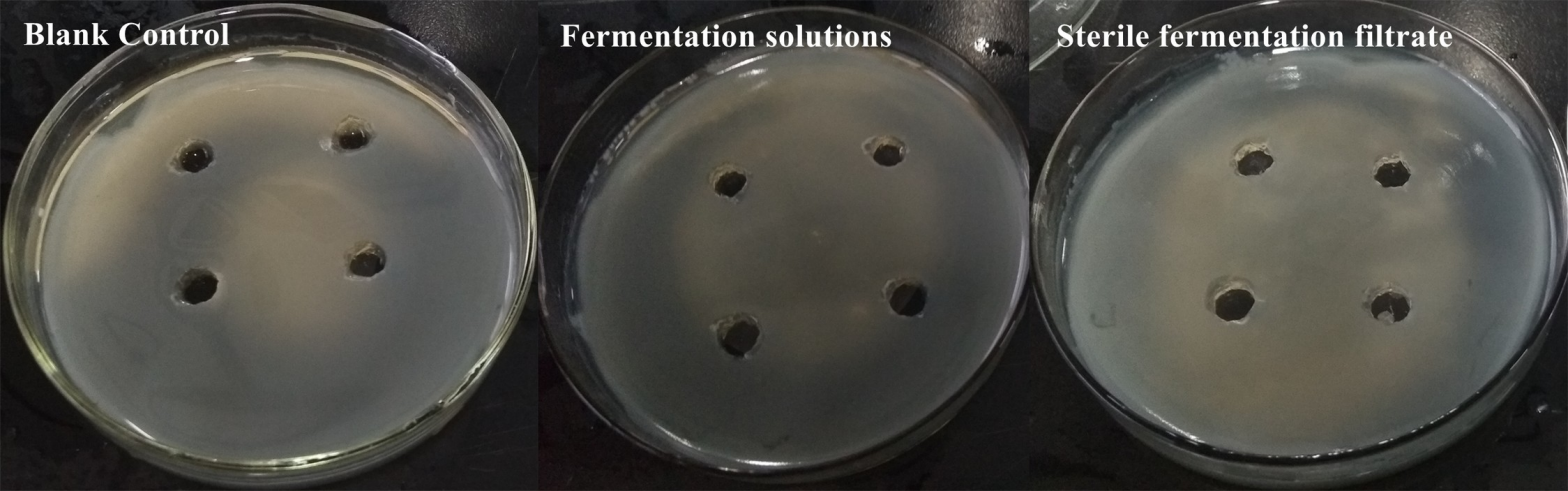
Antagonistic effect of fermentation solutions and sterile fermentation filtrate of CAB-L026.

To determine whether the antagonistic effect of the bacteria exhibiting biocontrol was caused by the bacteria itself or the secondary antimicrobial metabolites produced by the bacteria, the antagonistic effects of the nine strains with biocontrol effect on pathogenic bacteria of soft rot disease were measured. No inhibition zones were observed in the fermentation solutions and the sterile fermentation filtrates (Fig 3). The results showed that the antagonistic effect of the strain was not caused by the secondary antimicrobial metabolites, but by the strain itself.

### Molecular identification

**Fig 4:**
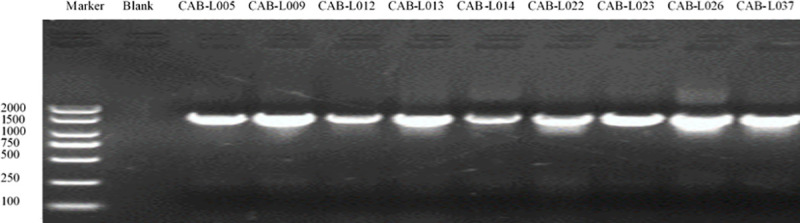
The results of the PCR amplification reaction system of biocontrol bacteria.

DNA was extracted from the nine samples that were not infected in the in vitro assay which were then amplified using the PCR method. The bands were clear and bright through the gel imaging system. The DNA length of about 1,500 bp was consistent with the expected size (Fig 4). The PCR products from the bacterial suspensions were sent to the company for sequencing.

According to the sequence analysis and comparison, CAB-L005, CAB-L013, and CAB-L037 shared the same sequence; CAB-L012, CAB-L02,3 and CAB-L026 shared the same sequence; CAB-L009, and CAB-L022 shared the same sequence; while CAB-L014 did not share a sequence with any other strains.

**Fig 5:**
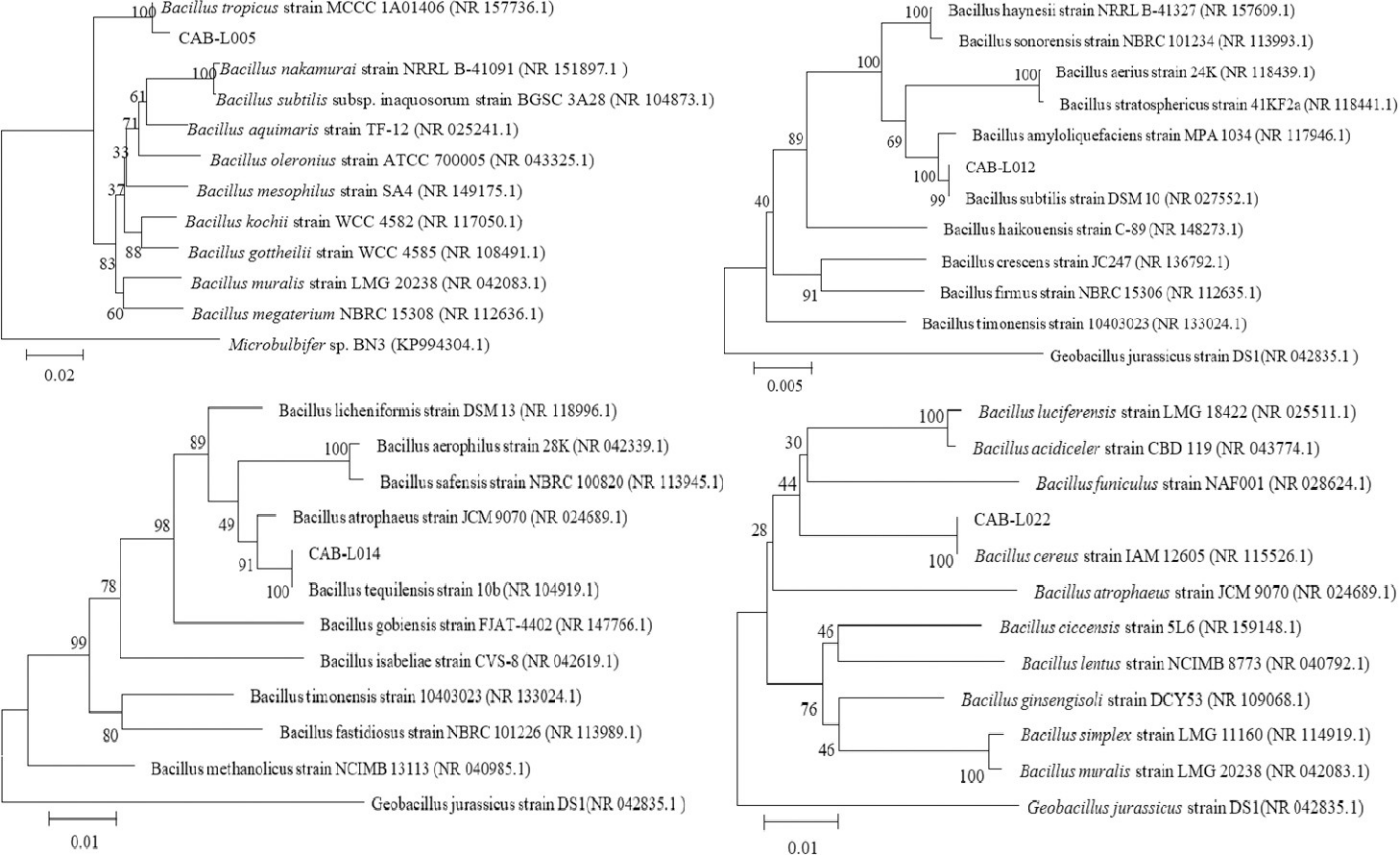
Construction of phylogenetic tree.

The results were analyzed by DNAMAN and compared with NCBI Blast online. The phylogenetic tree was constructed in MEGA5 (Fig 5). The results showed that CAB-L005 was most similar to *Bacillus tropicus* (100%), CAB-L012 was most similar to *Bacillus subtilis* (99%), CAB-L014 was most similar to *Bacillus tequilensis* (100%), and CAB-L022 was most similar to *Bacillus cereus* (100%).

### Morphology and physiological and biochemical characteristics of isolated bacteria

Following the *Manual for Systematic Identification of Common Bacteria* [16] and *Bergey’s Manual of Determinative Bacteriology* (8^th^ edition, 1984) [17], the morphological and physiological and biochemical characteristics of bacterial strains were compared to the strains CAB-L005, CAB-L012, CAB-L014, and CAB-L022. It was preliminarily determined that the four strains were of the *Bacillus* family which was consistent with the results of the molecular sequencing. Four biocontrol bacteria were inoculated on NA medium. The results of the microscopic observations and the main physical and chemical characteristics are shown in Table 1. It was found that the results of morphology, physiological and biochemical characteristics, and carbon and nitrogen sources of the strains varied.

**Table 1:** Morphology and physiological and biochemical characteristics.

		CAB-L005	CAB-L012	CAB-L014	CAB-L022
Strain		*Bacillus tropicus*	Bacillus subtilis	*Bacillus tequilensis*	Bacillus cereus
Colony morphology		Light yellow, smooth and wet	Beige white, rough with wrinkled skin	Beige white, smooth and wet	Light yellow, smooth and wet
Shape		Rod-shaped	Rod-shaped	Rod-shaped	Rod-shaped
Aerobic type		Facultative aerobic	Facultative aerobic	Facultative aerobic	Facultative aerobic
Flagella staining		+	+	+	+
Gram staining		+	+	+	+
Spore staining		+	+	+	+
Contact enzyme		+	+	+	+
Glucose acid production		+	+	+	+
Gelatin hydrolysis		+	+	+	+
Indole		-	-	-	+
Mannitol acid production		-	+	-	-
Nitrate reduction		+	+	+	+
Phenylalanine deaminase		-	-	-	-
Starch hydrolysis		+	+	+	+
Utilization of citrate salt		+	+	+	+
V-P		+	+	+	+
Carbon sources	Sucrose	+	+	+	+
	Citric Acid	-	-	-	-
	Glucose	+	+	+	+
Nitrogen source	Potassium nitrate	+	+	+	+
Salinity tolerance	2%	+	+	+	+
	5%	+	+	+	+
	7%	+	+	+	+
	10%	-	+	-	-

Note: +Positive, -Negative.

### Growth of isolated bacteria in response to different temperatures and pH values

**Fig 6:**
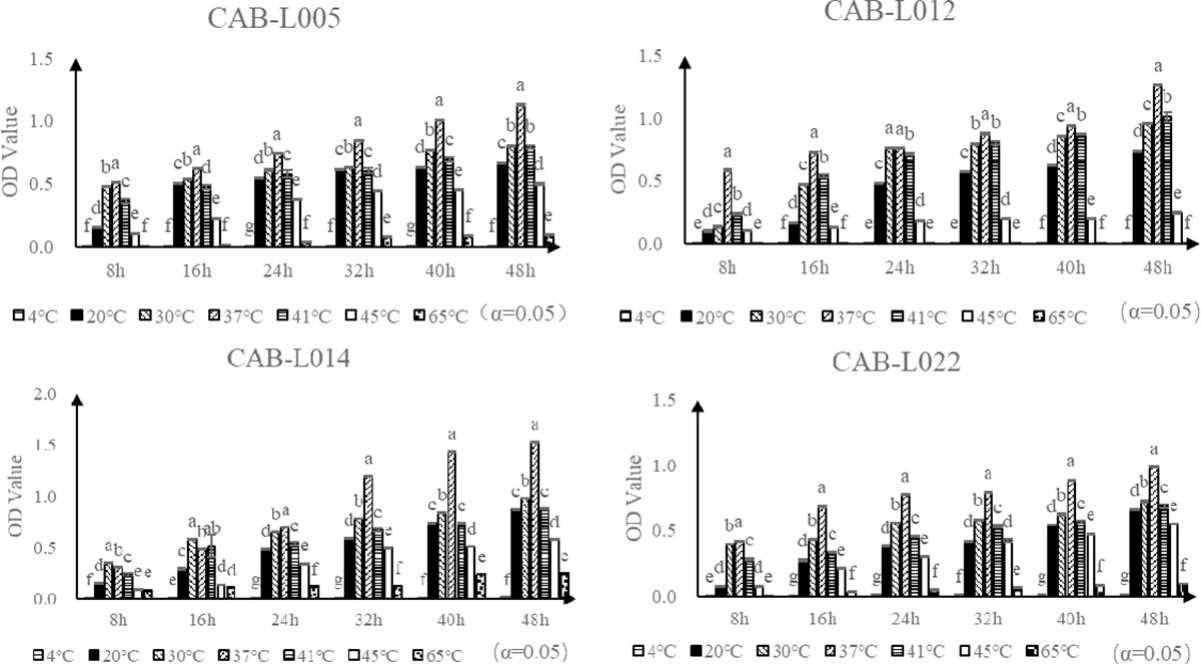
Growth of isolated bacteria at different temperatures. Note: Different lowercase letters on the curve indicate significant difference between groups (*P*<0.05).

The four isolated bacteria grew at different paces under different temperatures, with their growth increasing gradually from 4°C to 37°C at the same time point, and weakening gradually from 37°C to 65°C. Therefore, the peak temperature for growth was at 37°C, with growth decreasing when this temperature is increased or decreased. Thus, it can be concluded that the optimum growth temperature of the four strains is around 37°C. For heat tolerance, four strains can survive at 65°C but in very poor condition (Fig 6).

**Fig 7:**
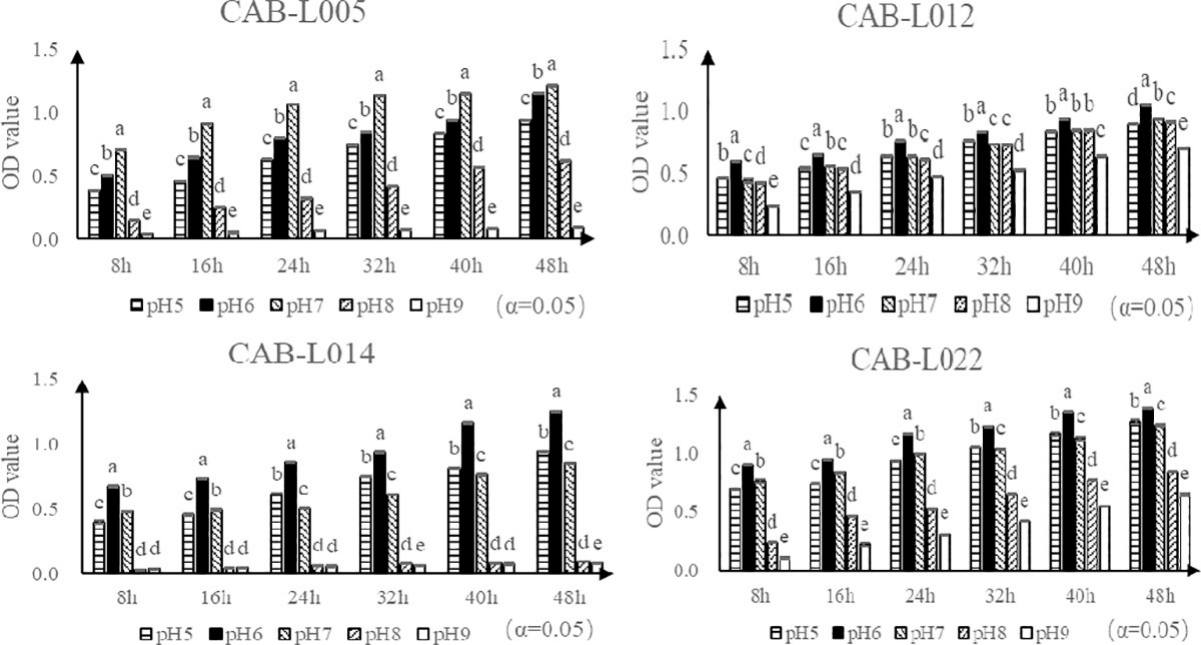
Growth of isolated bacteria at different pH values. Note: Different lowercase letters on the curve indicate significant difference between groups (*P*<0.05).

The four isolated bacteria grew at different paces at different pH values (5–9). It was observed that the optimum pH of CAB-L005 was 7, while the optimum pH of CAB-L012, CAB-L014, and CAB-L022 was 6 (Fig 7).

### In vivo field experiment

**Fig 8:**
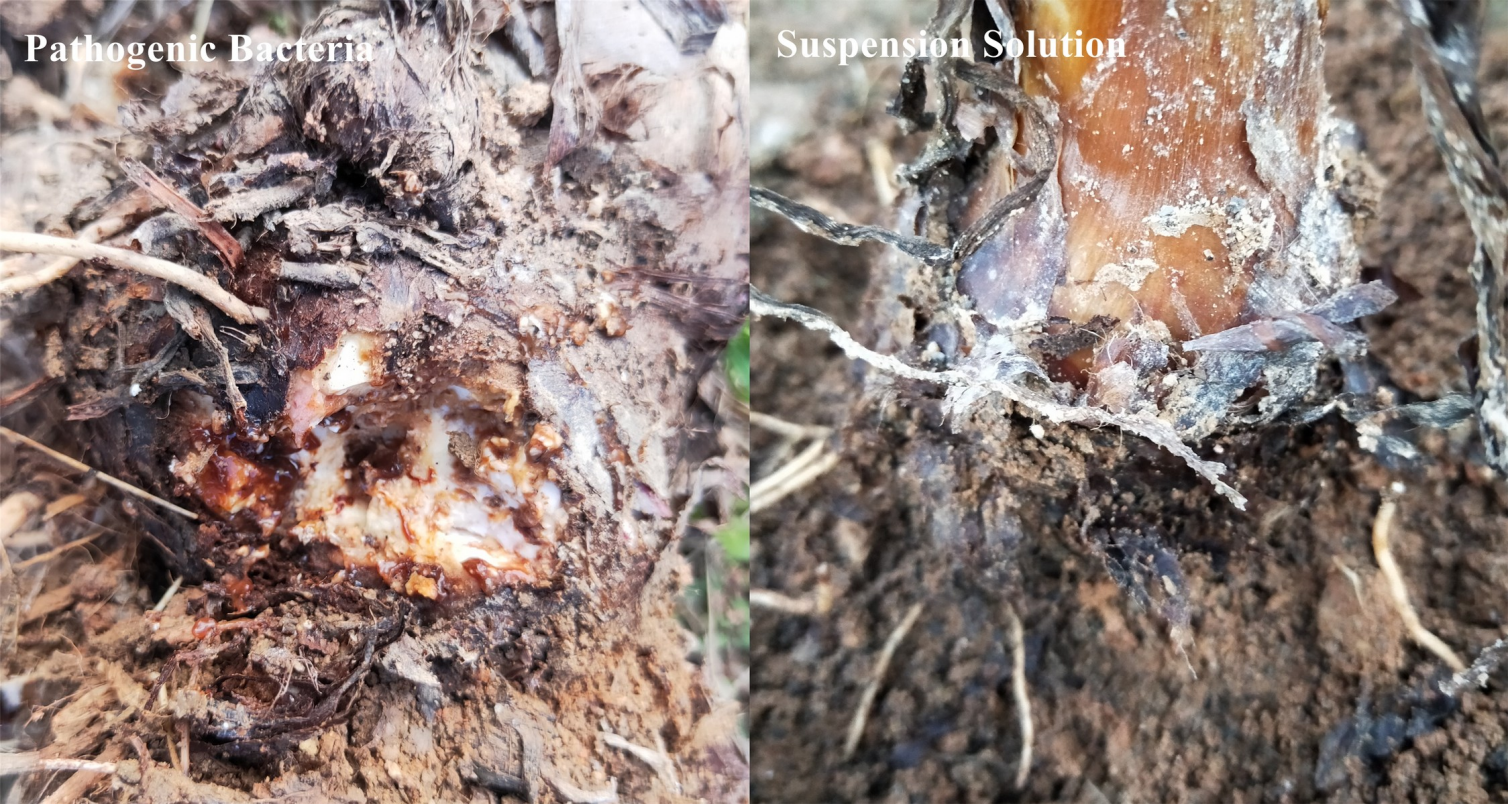
Biocontrol effect of plant corm.

Mixed bacterial suspensions were made with a 1:1 ratio of pathogenic bacteria of soft rot disease and biocontrol bacteria strain CAB-L005, CAB-L012, CAB-L014, or CAB-L022 (1.0 × 10^7^ CFU/mL). After 14 days, the observation results showed that the corm of the control plant was infected pathogenic bacteria with the wound becoming soft, deformed, rotten, and foul-smelling, while the corm with the mixed bacterial suspension had no obvious change (Fig 8).

## Discussion

In the long-term evolution process of organisms, various kinds of microorganisms around the plant rhizosphere are closely related to the plant itself; thus, some microorganisms can better inhibit the growth and reproduction of plant pathogens [18,19]. Many studies have found that biocontrol bacteria can prevent and control the spread of bacterial plant diseases by competing for space in plants, competing for nutrition, inducing plants to produce disease resistance, and other mechanisms [20,21]. The development of new pesticides that have bacteriostatic effects against microorganisms can effectively protect the soil environment and thus provide food safety. The development and application of new biofungicides are important prospective developments for pollution-free agricultural production [22,23]. Screening of fungi, bacteria, actinomycetes, and other microorganisms for inhibitory effect against pathogenic bacteria provides an important research basis for biological control of soil-borne diseases in farmlands [24,25]. In this experiment, 53 strains of bacteria were isolated and purified from the rhizosphere soil of areca taro plant in Fuding. Four different bacteria, namely, CAB-L005, CAB-L012, CAB-L014, and CAB-L022, were obtained from corm by screening in vitro. Molecular sequencing and physiological and biochemical experiments were also conducted. These were found to show high similarity with *Bacillus tropicus*, *Bacillus subtilis*, *Bacillus tequilensis*, and *Bacillus cereus*, respectively. It was found that these four strains exhibited biocontrol effect and showed value in biocontrol application. At present, there are few studies on the screening of biocontrol bacteria against pathogenic bacteria of soft rot disease in areca taro. This paper provides some references for the screening of biocontrol bacteria and the prevention and control of soft rot disease in areca taro.

There are many different microorganisms living in the rhizosphere soil of plants. Many studies have shown that the mechanisms of different biocontrol bacteria to improve the resistance of plants against diseases vary, with some working individually, and some working in synergy with other bacteria [26,27]. In addition, most biocontrol microorganisms survive in bacterial preparations. When they are used in the field, they are often affected by external factors such as soil humidity, temperature, pH value, crop growth status, and other microorganisms in the soil. It is a complicated process to apply experiments done in the laboratory to the field, and the biocontrol ability of the strains will change compared to experiments done research institutes [28]. In this study, only laboratory and small-scale field tests were carried out. Since the mechanism of soft rot disease in taro is complicated and affected by many factors, further studies will be needed on the inhibition mechanism, rules of plant colonization, application method of field control, and biosafety of the four biocontrol bacteria identified in this experiment.

The occurrence of plant diseases is often the result of infection by multiple pathogenic bacteria. It is found that taro disease is “complex infection-oriented,” composed of one dominant disease and multiple concomitant diseases. Therefore, in the process of control of soft rot disease in taro, we should explore a comprehensive control strategy that takes both pathogenic bacteria and pathogenic fungi into account to address both symptoms and root causes [29,30]. For example, factors such as whether there are disease-resistant varieties, disease occurrence rules, and disease cycles as well as whether controlling measures should be strengthened. Meanwhile, there are also other key measures to prevent and control disease, such as timely sowing and strengthening management in the field, enhancing resistance to disease, and crop rotation, to reduce the occurrence and harm caused by the disease [31].

## Supporting information

S1 Raw images (PDF)
