# Correction: Screening of biocontrol bacteria against soft rot disease of *Colocasia esculenta* (L.) *schott* and its field application

**DOI:** 10.1371/journal.pone.0314065

**Published:** 2024-11-14

**Authors:** Xiaofei Dong, Lu Fang, Zuyun Ye, Guangqiang Zhu, Qianyu Lai, Shengrong Liu

In Fig 2, the picture of CAB-L026 is incorrect. Please see the correct Fig 2 here.

**Fig 2:**
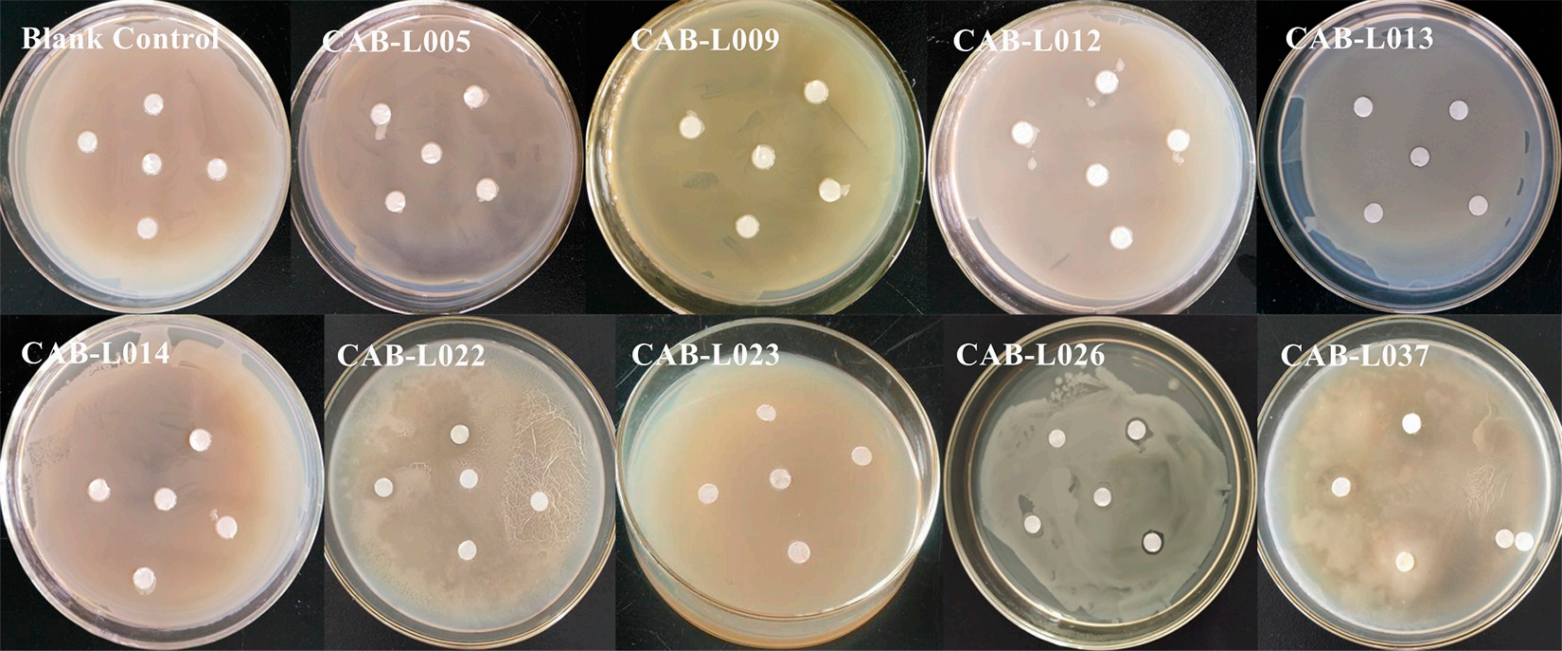
Antagonistic effect of plate.
