# Supplementary figures and images for: Screening of biocontrol bacteria against soft rot disease of Colocasia esculenta (L.) schott and its field application
Source: PLoS One. 2021 Jul 12;16(7):e0254070. doi: 10.1371/journal.pone.0254070 (PMC8274833; doi:10.1371/journal.pone.0254070)

Marker    Blank    CAB-L005    CAB-L009    CAB-L012    CAB-L013    CAB-L014    CAB-L022    CAB-L023    CAB-L026    CAB-L037    X

2000  
1500  
1000  
750  
500  
250  
100

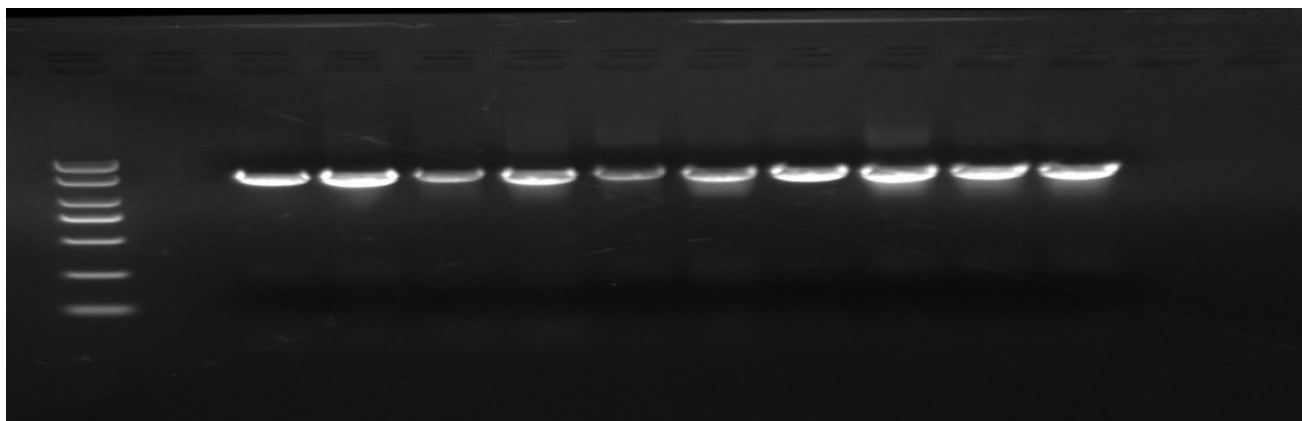

Supplement: S1 Raw images — (PDF) [file pone.0254070.s001.pdf]
